# The clinical characteristics and treatment response of patients with chronic obstructive pulmonary disease with low body mass index

**DOI:** 10.3389/fphar.2023.1131614

**Published:** 2023-07-13

**Authors:** Qing Song, Aiyuan Zhou, Ling Lin, Xueshan Li, Wei Cheng, Cong Liu, Yating Peng, Yuqin Zeng, Rong Yi, Yi Liu, Xin Li, Yan Chen, Shan Cai, Ping Chen

**Affiliations:** ^1^ Department of Respiratory and Critical Care Medicine, The Second Xiangya Hospital, Central South University, Changsha, Hunan, China; ^2^ Research Unit of Respiratory Disease, Central South University, Changsha, Hunan, China; ^3^ Diagnosis and Treatment Center of Respiratory Disease, Central South University, Changsha, Hunan, China; ^4^ Center of Respiratory Medicine, Xiangya Hospital, Central South University, Changsha, Hunan, China; ^5^ Department of Pulmonary and Critical Care Medicine, Zhuzhou Central Hospital, Zhuzhou, Hunan, China; ^6^ Division 4 of Occupational Diseases, Hunan Prevention and Treatment Institute for Occupational Diseases, Changsha, Hunan, China

**Keywords:** chronic obstructive pulmonary disease, body mass index, minimum clinically important difference, clinically important deterioration, exacerbation, mortality

## Abstract

**Background:** This study aimed to analyze the clinical characteristics and treatment response of patients with chronic obstructive pulmonary disease (COPD) with low body mass index (BMI).

**Methods:** In this cross-sectional study, we enrolled patients with stable COPD from the database setup by the Second Xiangya Hospital of Central South University. We classified the patients into three groups based on BMI: low-BMI (<18.5 kg/m^2^), normal-BMI (≥18.5 and <24.0 kg/m^2^), and high-BMI (≥24 kg/m^2^) groups. We defined clinically important deterioration (CID) as a COPD Assessment Test (CAT) score increase of ≥2 and minimum clinically important difference (MCID) as a CAT score decrease of ≥2 during 6 months of follow-up. We recorded the number of exacerbations and mortality during 1 year of follow-up.

**Results:** A total of 910 COPD patients were included with 144 (15.8%) patients in low-BMI, 475 (52.2%) in normal-BMI, and 291 (32.0%) in high-BMI groups. Patients with low BMI had worse pulmonary function, higher symptom scores, and exacerbations in the past year compared with normal- and high-BMI groups (*p* < 0.05). Logistic regression analysis revealed that age, Global Initiative for Chronic Obstructive Lung Disease grades 3 and 4, and hospitalizations in the past year were independent risk factors for patients with low BMI (*p* < 0.05). After 1 year of follow-up, patients with low BMI had higher mortality and number of hospitalizations. Patients with low BMI were more likely to attain CID and less likely to attain MCID compared with patients with high BMI (*p* < 0.05). In addition, patients with low BMI treated with long-acting β2-agonist (LABA)+long-acting muscarinic antagonist (LAMA) and LABA+LAMA+inhaled corticosteroid (ICS) were more likely to attain MCID than those treated with LABA+ICS and LAMA (*p* < 0.05).

**Conclusion:** COPD patients with low BMI had worse pulmonary function, higher symptom scores, and higher risk of future hospitalizations and mortality and were less likely to attain MCID and more likely to attain CID. It is worth noting that patients with low BMI treated with LABA+LAMA and LABA+LAMA+ICS were more likely to attain MCID than those treated with LABA+ICS and LAMA.

## Introduction

Chronic obstructive pulmonary disease (COPD) is the most common chronic respiratory disease. It has high morbidity and mortality and exerts huge burden on societies. COPD has become the third leading cause of death (GBD Chronic Respiratory Disease Collaborators, 2020). Thus, treatment and prevention are urgent.

Body mass index (BMI), calculated as the weight in kilograms divided by the square of the height in meters (kg/m^2^), is an important indicator to evaluate nutritional status. In fact, BMI plays an important role in the pathophysiology of COPD and is an independent prognostic factor for mortality and severity of COPD (Yang et al., 2010). Putcha et al. (2022) showed that COPD patients with low BMI (<20 kg/m^2^) had worse pulmonary function and higher risk of future severe exacerbation and mortality. In addition, low BMI (<20 kg/m^2^) is associated with higher risk of first acute COPD admission (Hunter et al., 2016). In fact, the BMI classification standard established by the World Health Organization is not completely suitable for the Chinese population. In China, BMI <18.5 kg/m^2^ is defined as underweight (Ran et al., 2007). A study from Taiwan demonstrated that overweight patients had a lower frequency of exacerbation in the past year compared with patients with normal BMI (18.5–23.9 kg/m^2^). However, patients with low BMI (<18.5 kg/m^2^) did not show a higher frequency of exacerbation compared with patients with normal BMI (Wei et al., 2017). Currently, the clinical characteristics and treatment response of COPD patients with low BMI have not been completely described in the Chinese population.

The Global Initiative for Chronic Obstructive Lung Disease (GOLD) documents recommend that the aim of COPD management is to reduce the symptoms and future risk of exacerbation and mortality. Currently, long-term medications including long-acting muscarinic antagonist (LAMA), long-acting β2-agonist (LABA)+inhaled corticosteroid (ICS), LABA+LAMA, and LABA+LAMA+ICS are the first choice for the treatment of patients with COPD (GOLD Executive Committee, 2023). However, it is unclear whether the treatment response differs among different inhalation therapies including LAMA, LABA+ICS, LABA+LAMA, and LABA+LAMA+ICS in COPD patients with low BMI.

Therefore, the purpose of this study was to analyze the clinical characteristics and treatment response of COPD patients with low BMI in the Chinese population and to explore the relationship between treatment response and different inhalation therapies in patients with low BMI.

## Patients and methods

### Study participants

This was a multicentric and cross-sectional study. All subjects were from the outpatient COPD database (Register number ChiCTR-POC-17010431) that includes the Second Xiangya Hospital of Central South University, Zhuzhou Central Hospital, the Hunan Prevention and Treatment Institute for Occupational Diseases, the First Attached Hospital of Shaoyang University, the Eighth Hospital in Changsha, and Longshan Hospital of Traditional Chinese Medicine (Hunan, China). The patients had been diagnosed with COPD between December 2016 and November 2021 according to the GOLD 2017 documents: the ratio of the forced expiratory volume in one second to forced vital capacity (FEV1/FVC) was <0.70 after inhaling a bronchodilator (Vogelmeier et al., 2017). Patients with asthma, lung cancer, pneumonia, bronchiectasis, tuberculosis, obstructive sleep apnea, diabetes, hormonal disorder, hypertension, and severe heart, liver, or kidney disease were excluded from this study.

This study was conducted in accordance with the Declaration of Helsinki and approved by the Ethics Committee of the Second Xiangya Hospital of Central South University (Hunan, China). All patients provided written informed consent.

### Data collection

Data including age, sex, education level, BMI, smoke history, FEV1 %pred, FEV1/FVC, GOLD grades, GOLD groups, COPD Assessment Test (CAT) scores, modified Medical Research Council (mMRC) scores, exacerbations and hospitalizations in the past year, and inhalation therapy regimens were collected at the patients’ first visit. At 6 months of follow-up, the CAT scores were recorded. The number of exacerbations, hospitalizations, and deaths was recorded during 1 year of follow-up.

### Study procedures

According to the BMI (weight in kilograms divided by the square of the height in meters) at their first visit, the patients were classified into three groups (Ran et al., 2007), namely, low-BMI (<18.5 kg/m^2^), normal-BMI (18.5–23.9 kg/m^2^), and high-BMI (≥24 kg/m^2^) groups. Then, the patients with low BMI were classified into the LAMA, LABA+ICS, LABA+LAMA, and LABA+LAMA+ICS subgroups based on the inhalation therapy they received at their first hospital visit.

### Treatment assessment

The minimum clinically important difference (MCID) and clinically important deterioration (CID) response rates, future exacerbation, and mortality were used to evaluate the effectiveness of the therapy. CID was defined as a CAT score increase of ≥2, while MCID was defined as a CAT score decrease of ≥2 during 6 months of follow-up (Kon et al., 2014).

### Variable definition

An exacerbation is COPD progression that requires antibiotics, oral corticosteroid, or hospitalization (Vogelmeier et al., 2017). According to the GOLD 2017 documents, patients with COPD assigned to group A show 0 to 1 exacerbation per year, no hospitalization, a CAT score of <10, and/or an mMRC score of 0–1. Group B shows 0–1 exacerbation per year, no hospitalization, a CAT score of ≥10, and/or an mMRC score of ≥2. Group C shows ≥2 exacerbations or ≥1 hospitalization per year, a CAT score of <10, and/or an mMRC score of 0–1. Group D shows ≥2 exacerbations or ≥1 hospitalization per year, a CAT score of ≥10, and/or an mMRC score of ≥2 (Vogelmeier et al., 2017). A current smoker has had a smoking exposure of ≥10 pack-years, while an ex-smoker has had an exposure of ≥10 pack-years but had not smoked for more than 6 months (Song et al., 2021).

### Statistical analysis

SPSS Statistics Version 26.0 (IBM, Armonk, NY, USA) and Free Statistics software version 1.7.1 (Beijing, China) were used for statistical analysis of the data. Continuous variables are expressed as the mean ± standard deviation or median and interquartile range (IQR). Continuous variables with a normal distribution and homogeneity of variance were analyzed with the analysis of variance; otherwise, non-parametric tests were used. The chi-squared test or Fisher’s exact test was used to analyze categorical variables. Propensity score matching (PSM) was conducted by using package R 2.15.3 (http://www.R-project.org). Adjusted odds ratio (aOR) and adjusted 95% confidence interval (a95% CI) were calculated by using logistic regression. A value of *p* < 0.05 was considered to be statistically significant.

## Results

### Clinical characteristics of the patients

**FIGURE 1:**
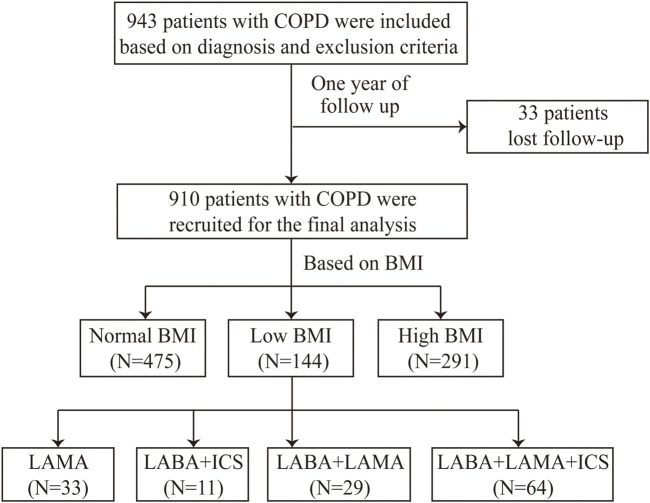
Flow chart. BMI, body mass index; COPD, chronic obstructive pulmonary disease; ICS, inhaled corticosteroid; LAMA, long-acting muscarinic antagonist; and LABA, long-acting β2-agonist.

A total of 910 patients with COPD were enrolled in this study (Figure 1). The mean age was 64.7 ± 8.3 years, and the majority were male (86.8%). The patients were assigned to low-BMI (15.8%), normal-BMI (52.2%), and high-BMI (32.0%) groups (Table 1).

**TABLE 1:** Clinical characteristics of the COPD patients.

Variables	Total (N = 910)
Age (years)	64.7 ± 8.3
Sex, n (%)	
Male	790 (86.8)
Female	120 (13.2)
Education level, n (%)	
Under junior high school	705 (77.5)
Over high school	205 (22.5)
BMI (kg/m^2^), n (%)	
<18.5	144 (15.8)
18.5–24	475 (52.2)
≥24	291 (32.0)
Smoke history, n (%)	
Never smoker	233 (25.6)
Ex-smoker	348 (38.2)
Current smoker	329 (36.2)
Smoking (packs/year) (median, IQR)	30 (5, 50)
Biofuel exposure, n (%)	
Yes	351 (38.6)
No	559 (61.4)
Pulmonary function (mean ± SD)	
FEV1 %pred	50.1 ± 20.1
FEV1/FVC	46.3 ± 12.5
GOLD grades, n (%)	
1	74 (8.1)
2	332 (36.5)
3	361 (39.7)
4	143 (15.7)
GOLD groups, n (%)	
A	132 (14.4)
B	427 (46.9)
C	82 (9.0)
D	269 (29.7)
CAT (mean ± SD)	15.3 ± 6.5
mMRC (median, IQR)	2 (1, 3)
Therapy, n (%)	
LAMA	240 (26.4)
LABA+ICS	82 (9.0)
LABA+LAMA	184 (20.2)
LABA+LAMA+ICS	348 (38.2)
Others^a^	56 (6.2)
Exacerbations in the past year (median, IQR)	1 (0, 2)
Exacerbations in the past year, n (%)	
0	396 (43.5)
1	211 (23.2)
≥2	303 (33.3)
Hospitalizations in the past year (median, IQR)	0 (0, 1)
Hospitalizations in the past year, n (%)	
0	637 (70.0)
≥1	273 (30.0)

^a^ Others including SAMA, SABA, SAMA+SABA, and no inhalation therapy.

BMI, body mass index; COPD, chronic obstructive pulmonary disease; CAT, COPD Assessment Test; FEV1, forced expiratory volume in one second; FVC, forced vital capacity; ICS, inhaled corticosteroid; LAMA, long-acting muscarinic antagonist; LABA, long-acting β2-agonist; mMRC, modified Medical Research Council; SAMA, short-acting muscarinic antagonist; SABA, short-acting β2-agonist.

As shown in Table 2, the COPD patients with low BMI had lower FEV1 %pred and FEV1/FVC and higher CAT scores, mMRC scores, number of exacerbations and hospitalizations in the past year compared with patients with normal BMI and high BMI (*p* < 0.05). In addition, the low-BMI group had a higher proportion of patients in GOLD grade 4, group D, exacerbations in the past year (≥2 times per year), and hospitalizations in the past year (≥1 time per year) and a lower proportion of GOLD grades 1–2 (*p* < 0.05).

**TABLE 2:** Clinical characteristics in different BMI groups of COPD patients.

Variables	Low BMI (N = 144)	Normal BMI (N = 475)	High BMI (N = 291)	*p*-values
Age (years)	65.8 ± 8.9 ^b^	65.0 ± 8.0 ^b^	63.6 ± 8.5	**0.012**
Sex, n (%)				0.550
Male	121 (84.0)	414 (87.2)	255 (87.6)	
Female	23 (16.0)	61 (12.8)	36 (12.4)	
Education, n (%)				**0.006**
Under junior high school	122 (84.7) ^b^	374 (78.7)	209 (71.8)	
Over high school	22 (15.3) ^b^	101 (21.3)	82 (28.2)	
Smoke history, n (%)				0.658
Never smoker	37 (25.7)	121 (25.5)	75 (25.8)	
Ex-smoker	61 (42.4)	173 (36.4)	114 (39.2)	
Current smoker	46 (31.9)	181 (38.1)	102 (35.1)	0.116
Smoke (packs/year), (median, IQR)	30 (7.9, 40.8)	35 (5, 50)	30 (3.2, 49.5)	
Biofuel exposure, n (%)				0.120
Yes	66 (45.8)	181 (38.1)	104 (35.7)	
No	78 (54.2)	294 (61.9)	187 (64.3)	
Pulmonary function (mean ± SD)				
FEV1 %pred	41.5 ± 16.6 ^a^ ^,^ ^b^	49.6 ± 20.1 ^b^	55.3 ± 20.1	<**0.001**
FEV1/FVC	42.3 ± 12.5 ^a^ ^,^ ^b^	45.8 ± 12.3 ^b^	49.2 ± 12.1	<**0.001**
CAT (mean ± SD)	17.5 ± 6.6 ^a^ ^,^ ^b^	15.5 ± 6.5 ^b^	13.9 ± 6.1	<**0.001**
mMRC (median, IQR)	2 (2, 3) ^a^ ^,^ ^b^	2 (1, 3)	2 (1, 3)	**0.001**
GOLD grades, n (%)				<**0.001**
1	3 (2.1)^a^ ^,^ ^b^	41 (8.6)	30 (10.3)	
2	32 (22.2)^a^ ^,^ ^b^	165 (34.7) ^a^	135 (46.4)	
3	67 (46.5) ^b^	197 (41.5)	97 (33.3)	
4	42 (29.2) ^a^ ^,^ ^b^	72 (15.2)	29 (10.0)	
GOLD groups, n (%)				<**0.001**
A	14 (9.7) ^b^	62 (13.1)	56 (19.2)	
B	55 (38.3) ^a^	238 (50.1)	134 (46.0)	
C	11 (7.6)	42 (8.8)	29 (10.0)	
D	64 (44.4)^a^ ^,^ ^b^	133 (28.0)	72 (24.8)	
Therapy, n (%)				0.504
LAMA	33 (22.9)	133 (28.0)	74 (25.4)	
LABA+ICS	11 (7.6)	38 (8.0)	33 (11.3)	
LABA+LAMA	29 (20.1)	94 (19.8)	61 (21.0)	
LABA+LAMA+ICS	64 (44.4)	176 (37.0)	108 (37.1)	
Others^c^	7 (5.0)	34 (7.2)	15 (5.2)	
Exacerbations in the past year (median, IQR)	1 (0, 3) ^a^ ^,^ ^b^	1 (0, 2)	1 (0, 2)	**0.003**
Exacerbations in the past year, n (%)				**0.008**
0	49 (34.0) ^a^ ^,^ ^b^	219 (46.1)	128 (44.0)	
1	29 (20.1)	106 (22.3)	76 (26.1)	
≥2	66 (45.9) ^a^ ^,^ ^b^	150 (31.6)	87 (29.9)	
Hospitalizations in the past year (median, IQR)	0 (0, 1) ^a^ ^,^ ^b^	0 (0, 1)	0 (0, 0)	**0.005**
Hospitalizations in the past year, n (%)				**0.011**
0	86 (59.7) ^a^ ^,^ ^b^	346 (72.8)	205 (70.4)	
≥1	58 (40.3) ^a^ ^**,**^ ^b^	129 (27.2)	86 (29.6)	

^a^ *p* < 0.05 *vs.* the normal-BMI group.

^b^ *p* < 0.05 *vs.* the high-BMI group. The bold *p*-values indicate statistical significance.

^c^ Others including SAMA, SABA, SAMA+SABA, and no inhalation therapy.

BMI, body mass index; COPD, chronic obstructive pulmonary disease; CAT, COPD Assessment Test; FEV1, forced expiratory volume in one second; FVC, forced vital capacity; ICS, inhaled corticosteroid; GOLD, Global Initiative for Chronic Obstructive Lung Disease; LAMA, long-acting muscarinic antagonist; LABA, long-acting β2-agonist; mMRC, modified Medical Research Council; SAMA, short-acting muscarinic antagonist; SABA, short-acting β2-agonist.

### Factors correlated with low BMI in patients with COPD

The univariate analysis showed several relative factors for COPD patients with low BMI, including FEV1/FVC, CAT scores, mMRC scores, education level, GOLD grades, and exacerbations and hospitalizations in the past year.

After adjusting for FEV1/FVC, CAT scores, mMRC scores, and exacerbations in the past year, logistic regression revealed that age (aOR = 1.025, a95% CI = 1.000–1.050, and *p* = 0.046), hospitalizations in the past year (aOR = 1.117, a95% CI = 1.006–1.360, and *p* = 0.041), GOLD grades 3 (aOR = 7.006, a95% CI = 1.861–26.378, and *p* = 0.004), and GOLD grades 4 (aOR = 15.296, a95% CI = 3.496–66.933, and *p* < 0.001) were independent risk factors for COPD patients with low BMI (Table 3).

**TABLE 3:** Multivariate analysis of relative factors for low BMI in COPD patients.

Variables	Univariate			Multivariate		
	OR	95% CI	*p*-values	aOR	a95% CI	*aP* values
Age	1.020	0.998–1.043	0.069	1.025	1.000–1.050	**0.046**
Sex						
Female	Reference					
Male	0.763	0.465–1.250	0.283			
Education level						
Under junior high school	Reference			Reference		
Over high school	0.574	0.354–0.932	**0.025**	0.595	0.358–0.988	**0.045**
Smoke history						
Never smoker	Reference					
Ex-smoker	1.126	0.720–1.760	0.603			
Current smoker	0.861	0.538–1.377	0.532			
Smoking (packs/year)	0.995	0.989–1.001	0.113			
Biofuel exposure						
No	Reference					
Yes	1.428	0.997–2.045	0.052			
Pulmonary function						
FEV1/FVC	0.968	0.954–0.983	**<0.001**			
GOLD grades						
1	Reference			Reference		
2	2.524	0.752–8.477	0.134	3.051	0.877–10.620	0.080
3	5.393	1.648–17.646	**0.005**	7.006	1.861–26.378	**0.004**
4	9.842	2.935–33.003	**<0.001**	15.296	3.496–66.933	**<0.001**
CAT	1.064	1.036–1.093	**<0.001**			
mMRC	1.354	1.127–1.627	**0.001**			
Exacerbations in the past year	1.061	1.014–1.100	**0.011**			
Hospitalizations in the past year	1.252	1.091–1.435	**0.001**	1.117	1.006–1.360	**0.041**

Multivariate analysis: Adjusting for FEV1/FVC, mMRC, CAT, and exacerbations in the past year. The bold *p*-values indicate statistical significance.

BMI, body mass index; CI, confidence interval; COPD, chronic obstructive pulmonary disease; CAT, COPD Assessment Test; FEV1, forced expiratory volume in one second; FVC, forced vital capacity; GOLD, Global Initiative for Chronic Obstructive Lung Disease; mMRC, modified Medical Research Council; aOR, adjusted odds ratio.

### Treatment response in different BMI groups after propensity score matching

After PSM, there were 96 (25.8%) patients in the low-BMI group, 183 (49.2%) patients in the normal-BMI group, and 93 (25.0%) patients in the high-BMI group. The baseline clinical characteristics showed no significant differences (Table 4).

**TABLE 4:** Clinical characteristics in different BMI groups of COPD patients after propensity score matching (PSM).

Variables	Total (N = 372)	PSM			*p*-values
		Low BMI (N = 96)	Normal BMI (N = 183)	High BMI (N = 93)	
Age (years)	65.3 ± 8.5	66.3 ± 9.2	65.5 ± 8.2	63.8 ± 8.5	0.128
Sex, n (%)					0.557
Male	310 (83.3)	83 (86.5)	152 (83.1)	75 (80.6)	
Female	61 (16.7)	13 (13.5)	31 (16.9)	18 (19.4)	
Education, n (%)					0.054
Under junior high school	313 (84.1)	82 (85.4)	160 (87.4)	71 (76.3)	
Over high school	58 (15.9)	24 (14.6)	23 (12.6)	22 (23.7)	
Smoke history, n (%)					0.232
Never smoker	110 (29.6)	24 (25.0)	56 (30.6)	30 (32.3)	
Ex-smoker	149 (40.1)	45 (46.9)	64 (35.0)	40 (43.0)	
Current smoker	113 (30.3)	7 (28.1)	63 (34.4)	23 (24.7)	
Smoke (packs/year), (median, IQR)	30 (0, 50)	30 (9.5, 40.8)	30 (0, 50)	30 (0, 50)	0.830
Biofuel exposure, n (%)					0.677
Yes	166 (44.6)	43 (44.8)	85 (46.4)	38 (40.9)	
No	206 (55.4)	53 (55.2)	98 (53.6)	55 (59.1)	
Pulmonary function (mean ± SD)					
FEV1 %pred	41.8 ± 15.7	41.1 ± 16.1	41.2 ± 15.8	43.8 ± 14.9	0.378
FEV1/FVC	42.0 ± 11.4	41.9 ± 13.2	41.1 ± 10.6	44.2 ± 10.7	0.097
GOLD group, n (%)					0.464
A	32 (8.6)	11 (11.5)	14 (7.7)	7 (7.5)	
B	184 (49.5)	40 (41.7)	100 (54.6)	44 (47.3)	
C	34 (9.1)	9 (9.4)	17 (9.3)	8 (8.6)	
D	122 (32.8)	36 (37.5)	52 (28.4)	34 (36.6)	
CAT (mean ± SD)	16.5 ± 6.5	16.7 ± 6.8	16.8 ± 6.6	15.7 ± 5.9	0.384
mMRC (median, IQR)	2 (2, 3)	2 (2, 3)	2 (2, 3)	2 (2, 3)	0.554
Therapy, n (%)					
LAMA	96 (25.8)	25 (26.0)	52 (28.4)	19 (20.4)	0.357
LABA+ICS	15 (4.0)	3 (3.1)	8 (4.4)	4 (4.3)	0.888
LABA+LAMA	33 (8.9)	10 (10.4)	13 (7.1)	10 (10.8)	0.497
LABA+LAMA+ICS	200 (53.8)	50 (52.2)	95 (51.9)	55 (59.1)	0.486
Others^a^	28 (7.5)	8 (8.3)	15 (8.2)	5 (5.4)	0.662
Exacerbations in the past year (median, IQR)	1 (0, 2)	1 (0, 3)	1 (0, 2)	1 (0, 2)	0.850
Hospitalizations in the past year (median, IQR)	0 (0, 1)	0 (0, 1)	0 (0, 1)	0 (0, 0)	0.420

^a^ Others including SAMA, SABA, SAMA+SABA, and no inhalation therapy.

BMI, body mass index; COPD, chronic obstructive pulmonary disease; CAT, COPD Assessment Test; FEV1, forced expiratory volume in one second; FVC, forced vital capacity; ICS, inhaled corticosteroid; GOLD, Global Initiative for Chronic Obstructive Lung Disease; LAMA, Long-acting muscarinic antagonist; LABA, long-acting β2-agonist; mMRC, modified Medical Research Council; PSM, propensity score matching; SAMA, short-acting muscarinic antagonist; SABA, short-acting β2-agonist.

As shown in Table 5, patients in the low-BMI group were less likely to attain MCID (53.9% *vs.* 69.6% and *p* < 0.05) and more likely to attain CID (27.0% *vs.* 12.0% and *p* < 0.05) compared with the high-BMI group. In addition, the future risk of hospitalizations and mortality in the low-BMI group was higher than in the normal- and high-BMI groups (*p* < 0.05).

**TABLE 5:** Treatment response in different BMI groups of COPD patients after propensity score matching.

Variables	Total (N = 372)	Low BMI (N = 96)	Normal BMI (N = 183)	High BMI (N = 93)	*p*-values
ΔCAT (median, IQR)	3 (−1, 7.2)	2 (−2, 7.0) ^b^	2 (−1.5, 7) ^b^	5 (0, 10)	**0.029**
MCID, n (%)					**0.040**
Yes	210 (58.3)	48 (53.9) ^b^	98 (54.7) ^b^	64 (69.6)	
No	150 (41.7)	41 (46.1) ^b^	81 (45.3) ^b^	28 (30.4)	
CID, n (%)					**0.022**
Yes	80 (22.2)	24 (27.0) ^b^	45 (25.1) ^b^	11 (12.0)	
No	280 (77.8)	65 (73.0) ^b^	134 (74.9) ^b^	81 (88.0)	
Exacerbations in the past year (median, IQR)	0 (0, 1)	0 (0, 1)	0 (0, 1)	0 (0, 1)	0.907
Exacerbations, n (%)					
0	258 (72.1)	61 (70.1)	130 (72.6)	67 (72.8)	
1	58 (16.2)	16 (18.4)	30 (16.8)	12 (13.0)	
≥2	42 (11.7)	10 (11.5)	19 (10.6)	13 (14.2)	
Hospitalizations in the past year (median, IQR)	0 (0, 0)	0 (0, 0)^a^ ^,^ ^b^	0 (0, 0)	0 (0, 0)	**0.007**
Hospitalizations, n (%)					**0.005**
0	307 (85.8)	66 (75.9) ^a^ ^,^ ^b^	157 (87.7)	85 (92.4)	
≥1	51 (14.2)	21 (24.1) ^a^ ^,^ ^b^	22 (12.3)	7 (7.6)	
Mortality, n (%)	14 (3.8)	9 (9.4) ^a^ ^,^ ^b^	4 (2.2)	1 (1.1)	**0.005**

^a^ *p* < 0.05 *vs.* the normal-BMI group.

^b^ *p* < 0.05 *vs.* the high-BMI group. The bold *p*-values indicate statistical significance. ΔCAT, CAT scores at 6 months of visit from baseline.

BMI, body mass index; COPD, chronic obstructive pulmonary disease; CAT, COPD Assessment Test; CID, clinically important deterioration; MCID, minimal clinically important difference.

### Treatment response among different inhalation therapies in patients with low BMI

Considering the poor treatment response of patients with low BMI, we further explored which inhalation drug would be better for patients with low BMI. A total of 137 patients with low BMI were classified into the LAMA (n = 33, 24.1%), LABA+ICS (n = 11, 8.0%), LABA+LAMA (n = 29, 21.2%), and LABA+LAMA+ICS (n = 64, 46.7%) subgroups after adjusting for sex, age, education level, smoke history, FEV1 %pred, CAT and mMRC scores, and exacerbations in the past year. Patients with low BMI treated with LABA+LAMA and LABA+LAMA+ICS were more likely to attain MCID than those treated with LAMA and LABA+ICS (*p* < 0.05). However, there were no significant differences in CID, future exacerbations, and mortality among the different inhalation therapies (Table 6; Table 7; Table 8).

**TABLE 6:** Treatment response among different inhalation therapies in patients with low BMI.

Variables	Total (N = 137)	LAMA (N = 33)	LABA+ICS (N = 11)	LABA+LAMA (N = 29)	LABA+LAMA+ICS (N = 64)	*p*-values
ΔCAT (median, IQR)	4 (−1, 8)	1.5 (−0.8, 6)	0 (−4, 6.5)	6 (2, 8)	4 (−1.8, 8.2)	0.146
MCID, n (%)						**0.016**
Yes	79 (61.2)	15 (45.5)	4 (36.4)	20 (76.9)	40 (67.8)	
No	50 (38.8)	18 (54.5)	7 (63.6)	6 (23.1)	19 (32.2)	
CID, n (%)						0.174
Yes	27 (20.9)	7 (21.2)	4 (36.4)	2 (7.7)	14 (23.7)	
No	102 (79.1)	26 (78.8)	7 (63.6)	24 (92.3)	45 (76.3)	
Exacerbations in the past year (median, IQR)	0 (0, 1)	0 (0, 0)	0 (0, 1)	0 (0, 1)	0 (0, 1)	0.626
Exacerbations, n (%)						0.873
0	86 (69.9)	24 (80.0)	7 (63.6)	18 (69.2)	37 (66.1)	
1	21 (17.1)	3 (10.0)	2 (18.2)	5 (19.2)	11 (19.6)	
≥2	16 (13.0)	3 (10.0)	2 (18.2)	3 (11.6)	8 (14.3)	
Hospitalizations in the past 1 year (median, IQR)	0 (0, 0)	0 (0, 0)	0 (0, 0)	0 (0, 1)	0 (0, 0)	0.175
Hospitalizations, n (%)						0.151
0	99 (80.5)	28 (93.3)	9 (81.8)	18 (69.2)	44 (78.6)	
≥1	24 (19.5)	2 (6.7)	2 (18.2)	8 (30.8)	12 (21.4)	
Mortality, n (%)	14 (10.2)	3 (9.1)	0 (0.0)	3 (10.3)	8 (12.5)	0.836

The bold *p*-values indicate statistical significance. ΔCAT, CAT score at the 6-month visit from baseline.

BMI, body mass index; CAT, COPD Assessment Test; CID, clinically important deterioration; MCID, minimal clinically important difference.

**TABLE 7:** Multivariate analysis for MCID in patients with low BMI.

Variables	MCID^a^				MCID^b^			
	OR (95%CI)	*p*-values	aOR (95%CI)	*aP*	OR (95%CI)	*p*-values	aOR (95%CI)	*aP*
				values				values
Therapy								
LAMA	Reference		Reference		1.46 (0.36–5.95)	0.599	1.71 (0.34–8.56)	0.512
LABA+ICS	0.69 (0.17–2.80)	0.599	0.58 (0.12–2.92)	0.512	Reference		Reference	
LABA+LAMA	4.00 (1.28–12.52)	**0.017**	3.95 (1.04–15.00)	**0.043**	5.83 (1.26–26.95)	0.024	6.78 (1.16–39.71)	**0.034**
LABA+LAMA+ICS	2.53 (1.05–6.07)	**0.038**	3.21 (1.10–9.38)	**0.033**	3.68 (0.96–14.13)	0.057	5.50 (1.09–27.73)	**0.039**
Age	1.02 (0.98–1.06)	0.438	1.01 (0.96–1.07)	0.653	1.02 (0.98–1.06)	0.438	1.01 (0.96–1.07)	0.653
Sex								
Male	Reference		Reference		Reference		Reference	
Female	1.72 (0.62–4.77)	0.299	2.48 (0.45–13.73)	0.298	1.72 (0.62–4.77)	0.299	2.48 (0.45–13.73)	0.298
Education level								
Under junior high school	Reference		Reference		Reference		Reference	
Over high school	1.72 (0.62–4.77)	0.299	2.20 (0.60–8.10)	0.238	1.72 (0.62–4.77)	0.299	2.20 (0.60–8.10)	0.238
Smoke history								
Never smoker	Reference		Reference		Reference		Reference	
Former smoker	0.81 (0.33–1.97)	0.635	1.47 (0.32–6.89)	0.623	0.81 (0.33–1.97)	0.635	1.47 (0.32–6.89)	0.632
Current smoker	0.96 (0.38–2.47)	0.940	1.70 (0.37–7.89)	0.495	0.96 (0.38–2.47)	0.940	1.70 (0.37–7.89)	0.495
Biofuel exposure								
No	Reference		Reference		Reference		Reference	
Yes	0.98 (0.48–2.00)	0.962	0.84 (0.32–2.18)	0.720	0.98 (0.48–2.00)	0.962	0.84 (0.32–2.18)	0.720
FEV1 %pred	0.99 (0.96–1.01)	0.177	0.98 (0.95–1.01)	0.270	0.99 (0.96–1.01)	0.177	0.98 (0.95–1.01)	0.270
CAT	1.12 (1.05–1.19)	**<0.001**	1.16 (1.07–1.25)	**<0.001**	1.12 (1.05–1.19)	**<0.001**	1.16 (1.07–1.25)	**<0.001**
mMRC	1.06 (0.75–1.49)	0.746	0.49 (0.28–0.87)	**0.016**	1.06 (0.75–1.49)	0.746	0.49 (0.28–0.87)	**0.016**
Exacerbations in the past year	1.01 (0.92–1.11)	0.806	1.01 (0.91–1.12)	0.908	1.01 (0.92–1.11)	0.806	1.01 (0.91–1.12)	0.908

MCID^a^- LAMA as the reference; MCID^b^- LABA+ICS as the reference; *aP* values, after adjusting for sex, age, education level, smoke history, biofuel exposure, CAT, mMRC, FEV1 %pred, and exacerbation in the past year. The bold *p*-values indicate statistical significance.

BMI, body mass index; CAT, COPD Assessment Test; CI, confidence interval; FEV1, forced expiratory volume in one second; ICS, inhaled corticosteroid; LAMA, long-acting muscarinic antagonist; LABA, long-acting β2-agonist; mMRC, modified Medical Research Council; MCID, minimal clinically important difference; aOR, adjusted odds ratio.

**TABLE 8:** Multivariate analysis for CID and future exacerbation in patients with low BMI.

Variables	CID				Exacerbation			
	OR (95%CI)	*p*-values	aOR (95%CI)	*aP*	OR (95%CI)	*p*-values	aOR (95%CI)	*aP*
				values				values
Therapy								
LAMA	Reference		Reference		Reference		Reference	
LABA+ICS	2.12 (0.48–9.37)	0.321	3.26 (0.57–18.5)	0.183	1.52 (0.36–6.48)	0.569	3.07 (0.53–17.7)	0.209
LABA+LAMA	0.31 (0.06–1.64)	0.168	0.37 (0.06–2.51)	0.311	1.63 (0.56–4.76)	0.372	1.05 (0.29–3.79)	0.946
LABA+LAMA+ICS	1.16 (0.41–3.23)	0.783	1.35 (0.38–4.78)	0.640	1.95 (0.78–4.85)	0.153	1.00 (0.33–3.07)	0.996
Age	0.98 (0.94–1.03)	0.464	0.98 (0.93–1.04)	0.547	1.04 (0.99–1.08)	0.09	1.02 (0.97–1.08)	0.401
Sex								
Male	Reference		Reference		Reference		Reference	
Female	0.58 (0.16–2.15)	0.418	0.49 (0.06–3.96)	0.500	1.37 (0.55–3.40)	0.498	2.48 (0.40–15.4)	0.329
Education level								
Under junior high school	Reference		Reference		Reference		Reference	
Over high school	0.35 (0.08–1.60)	0.176	0.35 (0.06–2.15)	0.259	0.32 (0.10–1.01)	0.052	0.37 (0.10–1.38)	0.140
Smoke history								
Never smoker	Reference		Reference		Reference		Reference	
Former smoker	1.46 (0.49–4.32)	0.492	1.14 (0.18–7.02)	0.889	1.50 (0.63–3.58)	0.364	2.64 (0.47–14.7)	0.268
Current smoker	1.03 (0.32–3.32)	0.962	0.78 (0.12–5.11)	0.800	0.87 (0.34–2.22)	0.764	1.09 (0.19–6.16)	0.919
Biofuel exposure								
No	Reference		Reference		Reference		Reference	
Yes	1.36 (0.58–3.19)	0.474	1.83 (0.57–5.89)	0.309	2.37 (1.17–4.82)	**0.017**	1.87 (0.74–4.72)	0.187
FEV1 %pred	1.01 (0.99–1.04)	0.352	1.01 (0.97–1.05)	0.683	0.98 (0.96–1.00)	0.117	1.00 (0.97–1.03)	0.979
CAT	0.87 (0.81–0.94)	**<0.001**	0.86 (0.78–0.94)	**0.002**	1.10 (1.04–1.17)	**0.001**	1.09 (1.01–1.17)	**0.021**
mMRC	0.85 (0.56–1.27)	0.420	1.31 (0.69–2.48)	0.408	2.03 (1.37–3.03)	**<0.001**	1.65 (0.97–2.82)	0.064
Exacerbations in the past year	1.00 (0.89–1.12)	0.952	1.05 (0.93–1.18)	0.412	0.95 (0.86–1.06)	0.381	0.90 (0.78–1.04)	0.149

*aP* values, after adjusting for sex, age, education level, smoke history, biofuel exposure, CAT, mMRC, FEV1 %pred, and exacerbation in the past year. The bold *p*-values indicate statistical significance.

BMI, body mass index; CAT, COPD Assessment Test; CI, confidence interval; CID, clinically important deterioration; FEV1, forced expiratory volume in one second; ICS, inhaled corticosteroid; LAMA, long-acting muscarinic antagonist; LABA, long-acting β2-agonist; mMRC, modified Medical Research Council; aOR, adjusted odds ratio.

## Discussion

BMI is used as an indicator to assess the nutritional status of patients and is related to the prognosis of the diseases including COPD (Girón et al., 2009; Lainscak et al., 2011; Chen et al., 2018). Therefore, to prevent and treat COPD patients more effectively, it is necessary to explore the relationship between BMI and COPD. We found that the proportion of low-BMI patients accounted for 15.8%, which was higher than that in previous studies. This might be associated with the socioeconomic conditions in the previous year in China. In addition, Putcha et al. (2022) found that baseline FEV1 %pred and FEV1/FVC were lower in patients with low BMI. In the present study, we also found that patients with low BMI had worse pulmonary function. Unlike the study of Wei et al. (2017), in our study, patients with low BMI had higher number of exacerbations and hospitalizations in the past year. This difference might be because we excluded comorbidities including cardiovascular disease, hypertension, diabetes, and dyslipidemia from this study. Indeed, overweight has been linked to a better prognosis in patients with various chronic diseases, especially cardiovascular diseases, a phenomenon that has been termed the obesity paradox (Lavie et al., 2009). Furthermore, several independent risk factors for COPD patients with low BMI were identified including age, GOPD grades, and the number of hospitalizations in the past year.

Symptoms including cough, phlegm, chest tightness, dyspnea, and sleep impairment confer huge burdens to patients (Miravitlles and Ribera, 2017). In this study, CAT scores served as an indicator to assess whether the symptoms improved or deteriorated during the 6 months of follow-up. In fact, previous systematic reviews have confirmed the reliability and validity of CAT scores and have concluded that the tool is responsive to interventions. Furthermore, the correlation between CAT and St George’s Respiratory Questionnaire (SGRQ) scores is typically quite high, which has also been demonstrated (Gupta et al., 2014). Two or more patient-reported outcome measures such as CAT, SGRQ, and Evaluating Respiratory Symptoms are more suitable for randomized controlled trials. A single CAT score for assessing symptoms is more operable in real-world clinical practice and has been used in previous studies (Buhl et al., 2018; Cheng et al., 2021; Lin et al., 2023).

In addition, exacerbation and mortality are important events of deterioration in patients with COPD (Flattet et al., 2017; Dong et al., 2020). Reducing the symptoms and risk of exacerbation and mortality are the main goals in the treatment of COPD patients. In the present study, patients with low BMI had a higher future risk of hospitalizations and mortality during 1 year of follow-up. The CAT score is responsive to short-term changes in patients with COPD, including MCID and CID, indicating whether the symptoms have improved and deteriorated (Kon et al., 2014). Our study is the first to demonstrate that COPD patients with low BMI had a lower MCID response rate and a higher CID response rate compared with high-BMI patients. Overall, the patients with low BMI had worse treatment response.

Inhalation drugs are the main treatment used to reduce symptoms and risk of exacerbation and mortality and to improve the health status of patients with COPD. Currently, LAMA, LABA+LAMA, LABA+ICS, and LABA+LAMA+ICS are the most used treatment regimens (Nici et al., 2020; Song et al., 2021). We have previously found that patient with low BMI had a poor treatment response, but which inhalation drug would be better for low-BMI patients was uncertain. Our study is the first to show that patients with low BMI treated with LABA+LAMA and LABA+LAMA+ICS are more likely to attain MCID than those treated with LABA+ICS and LAMA. However, there were no significant differences in future exacerbations and mortality among different inhalation therapies in patients with low BMI. This finding is different from the ETHOS/KRONOS study (Ferguson et al., 2018; Singh et al., 2022), perhaps because our core focus was COPD patients with low BMI patients. Taken together, our findings indicate that it might be more appropriate to provide LABA+LAMA or LABA+LAMA+ICS as the initial inhalation therapy for COPD patients with low BMI.

This study has limitations. As recommended by the GOLD documents, non-pharmacological treatment including pulmonary rehabilitation, vaccination, and oxygen therapy is another method to reduce the symptoms and future risk of exacerbations for stable COPD patients and was not accounted for in this study. In fact, we have previously found that relatively few patients with COPD were receiving non-pharmacological treatment (Zeng et al., 2020). So, we do not believe that non-pharmacological treatment would have had a significant impact on the results of this study. In addition, this was a real-world and cross-sectional study. We included as many patients as possible who had completed the 1-year follow-up to obtain the data on exacerbation, mortality, and CAT scores. Therefore, we did not use power analysis to determine the sample size.

## Conclusion

COPD patients with low BMI had worse pulmonary function and higher symptom scores and number of exacerbations in the past year. Several independent risk factors for patients with low BMI were identified including age, GOLD grades, and hospitalizations in the past year. In addition, patients with low BMI had a higher risk of future hospitalizations and mortality and were less likely to attain MCID and more likely to attain CID. It was worth noting that patients with low BMI treated with LABA+LAMA and LABA+LAMA+ICS were more likely to attain MCID than those treated with LABA+ICS and LAMA.

## Data Availability

The raw data supporting the conclusion of this article will be made available by the authors, without undue reservation.

## Abbreviations

aOR, adjusted odds ratio; BMI, body mass index; COPD, chronic obstructive pulmonary disease; CAT, COPD Assessment Test; CID, clinically important deterioration; CI, confidence interval; FEV1, forced expiratory volume in one second; FVC, forced vital capacity; GOLD, Global Initiative for Chronic Obstructive Lung Disease; ICS, inhaled corticosteroid; IQR, interquartile range; LAMA, long-acting muscarinic antagonist; LABA, long-acting β2-agonist; mMRC, modified Medical Research Council; MCID, minimum clinically important difference; PSM, propensity score matching; and SGRQ, St George’s Respiratory Questionnaire.
